# Soil bacterial and fungal community dynamics in relation to *Panax notoginseng* death rate in a continuous cropping system

**DOI:** 10.1038/srep31802

**Published:** 2016-08-23

**Authors:** Linlin Dong, Jiang Xu, Guangquan Feng, Xiwen Li, Shilin Chen

**Affiliations:** 1 Institute of Chinese Materia Medica, China Academy of Chinese Medical Sciences, Beijing 100700, China; 2 Wenshan Prefecture Sanqi Science and Technology Research Institute, Sanqi Research department, Yunnan province 663000, China

## Abstract

Notoginseng (*Panax notoginseng*), a valuable herbal medicine, has high death rates in continuous cropping systems. Variation in the soil microbial community is considered the primary cause of notoginseng mortality, although the taxa responsible for crop failure remains unidentified. This study used high-throughput sequencing methods to characterize changes in the microbial community and screen microbial taxa related to the death rate. Fungal diversity significantly decreased in soils cropped with notoginseng for three years. The death rate and the fungal diversity were significantly negatively correlated, suggesting that fungal diversity might be a potential bioindicator of soil health. Positive correlation coefficients revealed that Burkholderiales, Syntrophobacteraceae, *Myrmecridium*, *Phaeosphaeria*, *Fusarium*, and *Phoma* were better adapted to colonization of diseased plants. The relative abundance of *Fusarium oxysporum* (*R* = 0.841, *P* < 0.05) and *Phaeosphaeria rousseliana* (*R* = 0.830, *P* < 0.05) were positively associated with the death rate. *F. oxysporum* was a pathogen of notoginseng root-rot that caused seedling death. Negative correlation coefficients indicated that Thermogemmatisporaceae, Actinosynnemataceae, Hydnodontaceae, Herpotrichiellaceae, and *Coniosporium* might be antagonists of pathogens, and the relative abundance of *Coniosporium perforans* was negatively correlated with the death rate. Our findings provide a dynamic overview of the microbial community and present a clear scope for screening beneficial microbes and pathogens of notoginseng.

Notoginseng (*Panax notoginseng*) is known for its therapeutic effects and is recognized as a highly valuable ingredient in medicinal products^1^,^2^. Notoginseng is a perennial plant cultivated in fixed plots with a continuous cropping (CC) pattern, which reduces tuber quality and yield^3^,^4^. Obstacles to replanting are prevalent among the *Panax* species, and replanting could fail because of high seedling death rates^5^. Soil conditions must be improved by many years of crop rotations before notoginseng can be replanted, and arable soils for notoginseng cultivation are becoming scarce.

Microbial diversity and community composition significantly affect agricultural soil productivity, plant growth, and crop quality^6^,^7^. Variation in the diversity and composition of the microbial community is believed to be related to changes in biotic and abiotic factors, such as cropping systems, plant species, and soil properties^8^,^9^. CC systems affect soil microbial diversity and community composition, thereby exerting significant negative impacts on soil productivity and health^6^,^10^. Understanding soil microbial communities is necessary to facilitate soil improvement under CC; however, few studies have examined the changes in bacterial and fungal communities in CC.

Changes in the diversity and composition of soil microorganisms can disrupt ecosystem function, balance, and health; in turn, such phenomena negatively affects soil productivity and ultimately leads to plant death^11^,^12^. The diversity and composition of soil microbial communities are closely related to the soil environmental characteristics, indicating that they could serve as sensitive bioindicators of soil health^13^,^14^. A number of bacterial and fungal taxa are associated with agricultural practices, and these taxa are potentially correlated with ecosystem metabolic processes^15^,^16^,^17^. However, few soil microbial groups related to the plant death rate index have been reported for CC.

Fungal communities are responsible for various soil-borne diseases in continuous peanut (*Arachis hypogaea* L.) cropping systems^10^. Certain fungal taxa might act as antagonists or pathogens of notoginseng under CC, but these fungal groups are yet to be identified. In notoginseng, root rot is the primary fungal disease caused by soil-borne pathogens, typically affecting 5–20% (even up to 70% in several reports) of the plants^18^,^19^. Root rot is caused by various pathogens, including *Fusarium* and *Phoma* spp., which are the main disease agents of notoginseng^20^,^21^. Despite the fact that these pathogens cause notoginseng root rot, minimal data are available on the presence of these groups in CC.

In the present study, we used high-throughput sequencing analysis of 16S and 18S rRNA genes to assess the variation of diversity and composition in soil microbial communities. We then analyzed the correlations between these communities and seedling death rate. Our results offered better insight into notoginseng death in relation to the rhizosphere microbial community. These data provides potentially useful information for soil bioremediation.

## Results

### Seedling death rates

**Figure 1:**
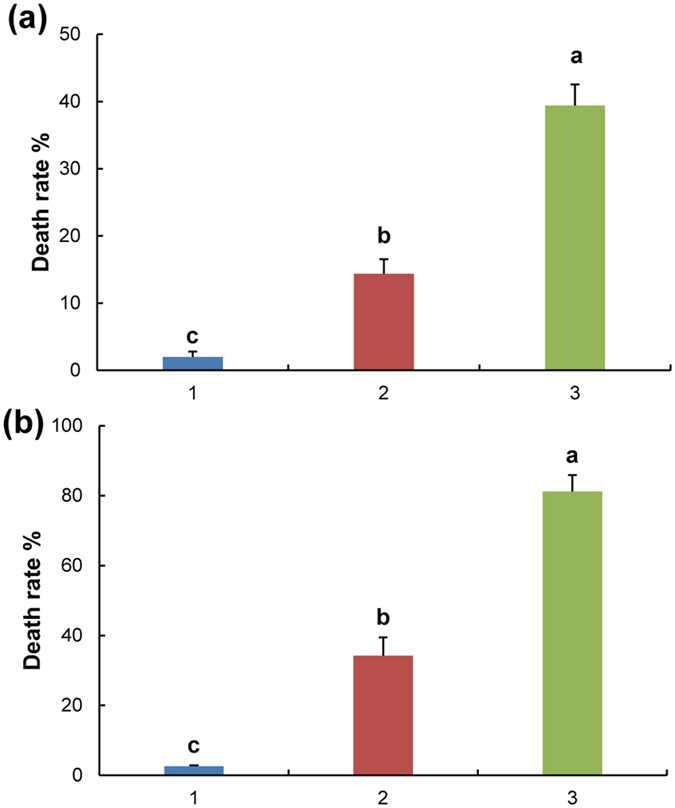
Notoginseng death rates under continuous cropping systems in 2012. (**a**) Death rates of notoginseng under continuous cropping for 1, 2 and 3 years. (**b**) Notoginseng death rates under replanted continuous cropping for 1, 2 and 3 years. CC, continuous cropping; RCC, replanted continuous cropping after rotation. All values are indicated as the mean ± SE (*n* = 3). Bars with different letters denote significant differences between different years (1, 2, and 3) of notoginseng cultivation at α = 0.05.

The death rates of notoginseng seedlings were 2.0–39.4% and 2.6–81.2% in the continuous cropping (CC) and replanted continuous cropping (RCC) systems, respectively (Fig. 1). A higher death rate was observed under RCC systems after rotation than under CC systems (Fig. 1; Supplementary Table S1).

### Ratio of fungi to bacteria

**Figure 2:**
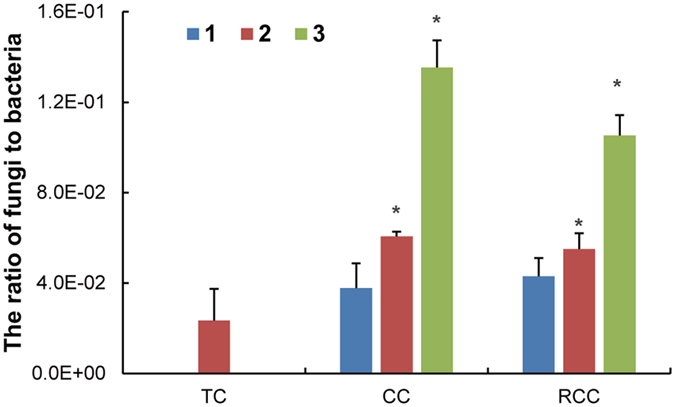
Ratio of fungi to bacteria in the soils. TC, traditional cropping; CC, continuous cropping; RCC, replanted continuous cropping after rotation. All values are indicated as the mean ± SE (*n* = 3). Asterisks denote significant differences between TC and notoginseng cultivation in the ratio of fungi to bacteria at *P* < 0.05.

The ratio of fungi to bacteria showed increasing trends in the soils of CC and RCC, respectively, compared to those of traditional cropping (TC) system soils (Fig. 2). The ratio was significantly higher in the soils of CC2, CC3, RCC2, and RCC3 compared to that of TC.

### Taxonomic diversity and their relationships with notoginseng death rates

**Figure 3:**
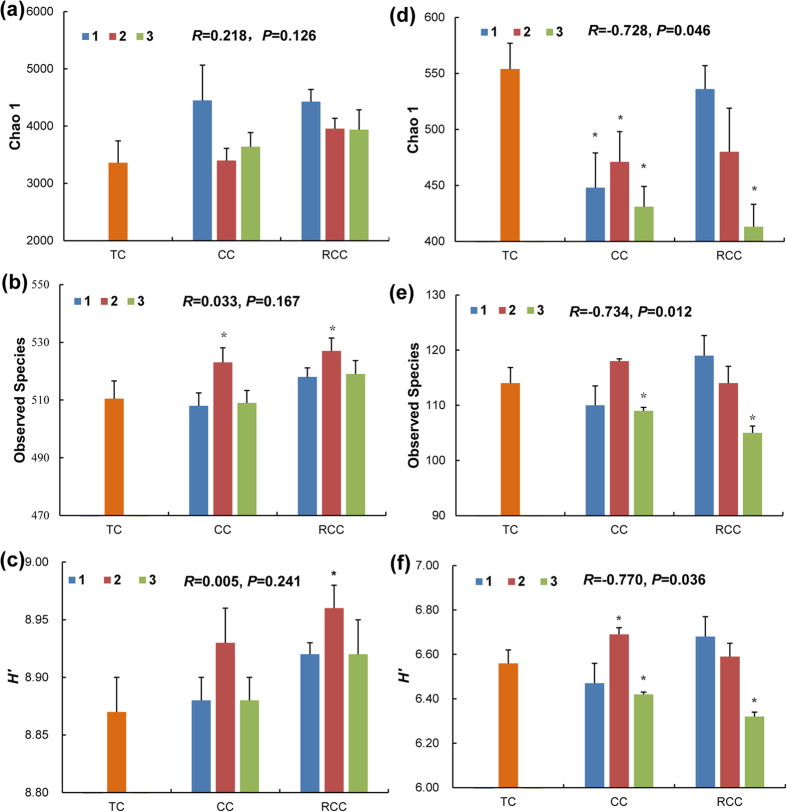
Bacterial and fungal diversity in traditional cropping and notoginseng cropping soils. (**a**–**c**) represent the Chao 1, observed species, and *H′* of bacterial community, respectively; (**d**–**f**) stand for the Chao1, observed species, and *H′* of fungal community, respectively. *H′*, Shannon diversity index; TC, traditional cropping (control); CC and RCC represent continuous cropping and replanted continuous cropping, respectively. *R* stands for the relationship between the diversity and notoginseng death rate based on the Pearson’s correlation analyses. All values are indicated as the mean ± SE (*n* = 3). Asterisks denote significant differences between the TC and notoginseng cultivation in the microbial diversity at *P* < 0.05.

A total of 60,495 bacterial sequences were obtained from 21 soil samples, of which 38,372 could be classified, for a mean of 1827 classifiable sequences per sample (range: 855–2906; dominant length: 205–231 bp; Supplementary Table S2). The values of Chao1 and Shannon diversity (*H′* ) were higher in CC and RCC soils than those in TC soil using a 3% dissimilarity cutoff (Fig. 3). Compared to that in TC, the observed species demonstrated an increasing trend in RCC and CC2 soils, whereas the observed species was lower in CC1 and CC3 soils. Pearson’s correlation analysis indicated that the death rate and the bacterial diversity index are not significantly related.

For fungi, we obtained 29,415 classifiable sequences with an average of 1400 sequences per soil sample (range: 300–2554; dominant length: 221–254 bp; Supplementary Table S2). Compared with those in TC, the value of Chao 1 decreased by 3.3–25.4% in CC and RCC soils; the observed species and *H′* of the fungal community exhibited decreasing trends in CC1, CC3, and RCC3 soils (Fig. 3). The value of Chao1, observed species, and *H′* of the fungal community significantly decreased in the soils continuously cropped with notoginseng for three years. Pearson’s correlation analysis revealed that the death rate was negatively related to the fungal diversity index (*P* < 0.05).

### Variation in bacterial community composition

**Figure 4:**
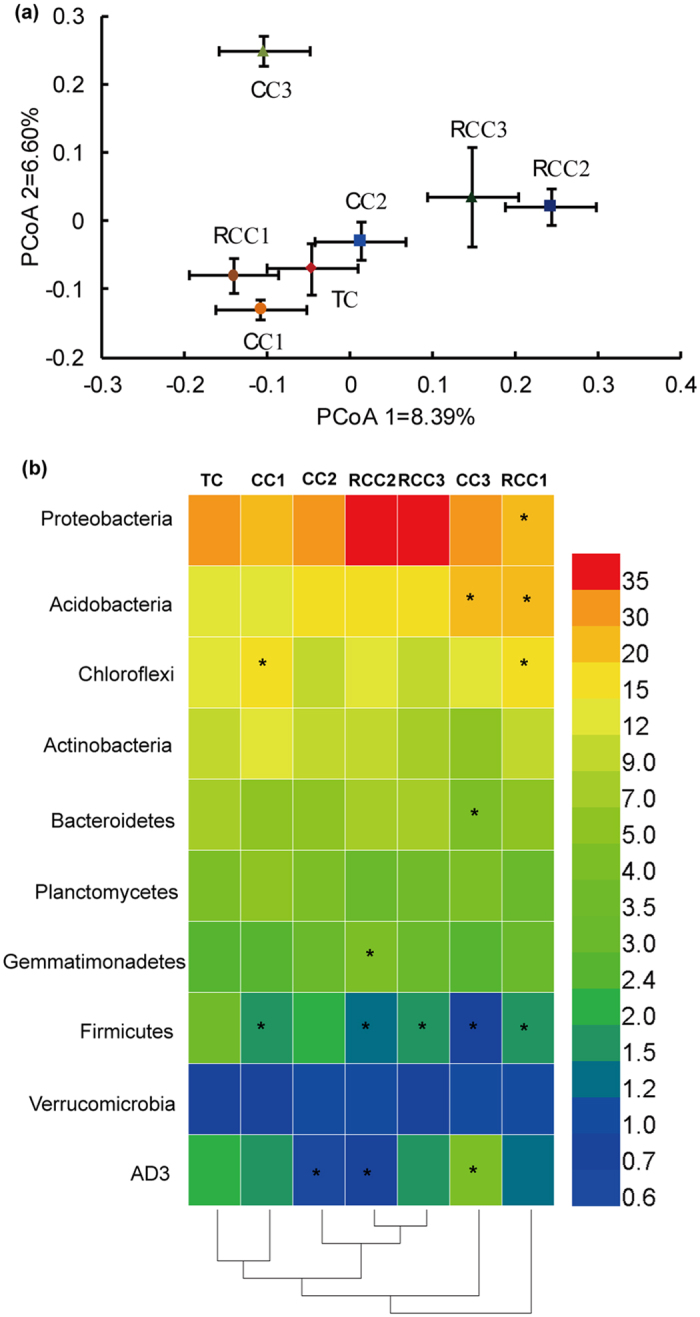
Changes of bacterial communities in notoginseng cropping and traditional cropping soils. (**a**) PCoA ordination plots display the relatedness of samples that were separated using Unweighted UniFrac distances of classified 16S rRNA gene sequences. (**b**) Heat map showing major bacterial phyla with average relative abundance >0.6% in all samples. Clustering on the *x*-axis is based on bacterial composition of the samples. Data are mean values of *n* = 3; asterisks denote significant differences between the TC and notoginseng cultivation in the relative abundance of soil bacterial groups at *P* < 0.05.

PCoA ordination revealed that bacterial communities differed between notoginseng cropping and TC soils (Fig. 4a). The first principal component (8.39% contribution) differentiated the bacterial communities in TC soils from those in RCC2 and RCC3 soils. Bray-Curtis distance matrix analysis indicates that the bacterial community composition of TC significantly differed from those of RCC2 and RCC3 (Supplementary Fig. S1a). The second principal component axis (6.60% contribution) showed that bacterial communities in CC3 varied from those in CC1 and CC2.

According to the linear discriminant analysis (LDA) effect size (LEfSe), 4, 12, and 24 groups were enriched in the soils of TC, CC, and RCC, respectively (Supplementary Fig. S2a). These groups mainly belonged to three phyla, namely, Chloroflexi, Actinobacteria, and Acidobacteria. A hierarchical dendrogram was used to compare the relative abundance of major bacterial taxa at the phylum level (Fig. 4b). Compared to that in TC, the relative abundance of Proteobacteria exhibited an increasing trend from 5.2% to 15.6% (except for CC1 and RCC1), and that of Acidobacteria increased from 2.2% to 51.9% in all CC soils. The relative abundance of Chloroflexi, Actinobacteria, Planctomycetes, Gemmatimonadetes, and AD3 fluctuated in CC and RCC soils in comparison with those in TC. The relative abundance of Bacteroidetes and Firmicutes decreased by 9.1–46.3% and 33.5–77.4%, respectively, under CC compared with that under TC. The low relative abundance of bacterial communities (<0.6%) mainly decreased and sometimes even completely disappeared (Supplementary Table S3). The relative abundance of WPS-2, Chlorobi, Fibrobacteres, Tenericutes, and Elusimicrobia decreased in notoginseng cropping soils compared to those in TC soils.

### Fluctuations in fungal community composition

**Figure 5:**
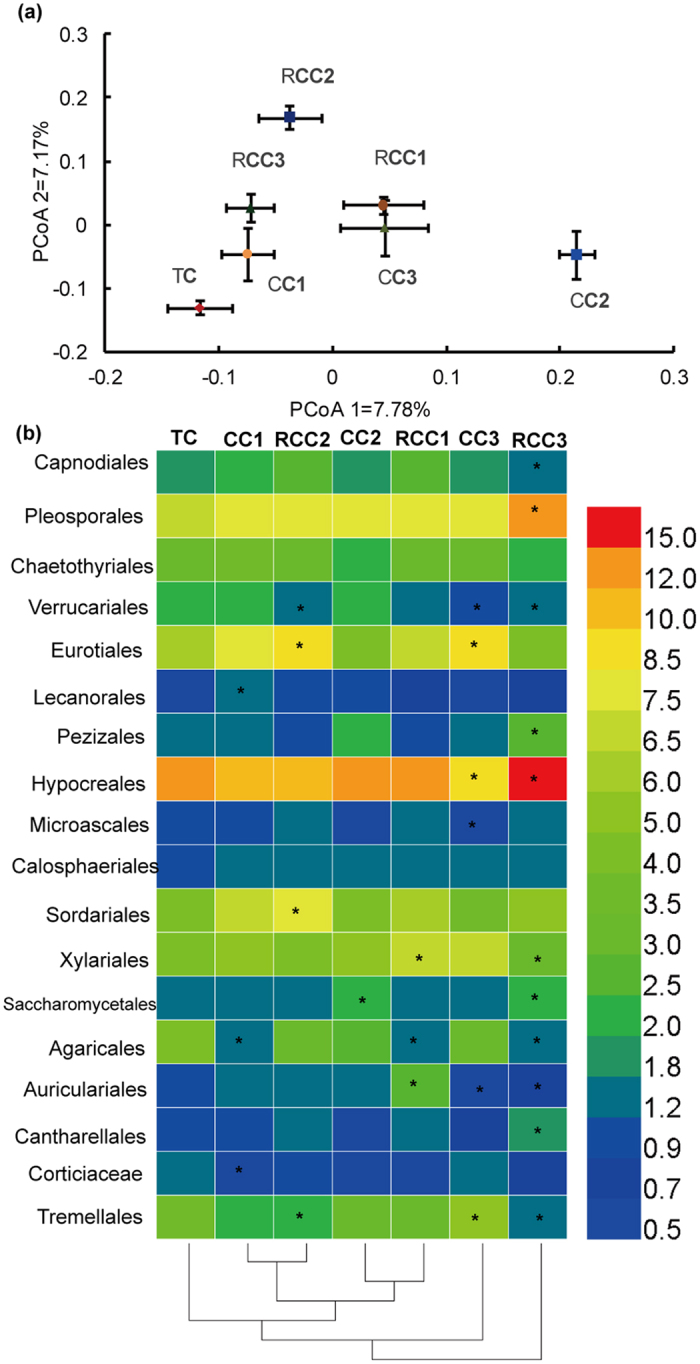
Changes in fungal communities in notoginseng cropping and traditional cropping soils. (**a**) PCoA ordination plots show the relatedness of samples that were separated using Unweighted UniFrac distances of classified 18S rRNA gene sequences. (**b**) Heat map shows major fungal orders with average relative abundance >0.5% in all samples. Clustering on the *x*-axis is based on fungal composition of the samples. Data are mean values of *n* = 3; asterisks denote significant differences between the traditional cropping and notoginseng cultivation in the relative abundance of soil fungal taxa at *P* < 0.05.

PCoA ordination revealed that fungal communities differed between notoginseng cropping and TC soils (Fig. 5a). The first principal component (7.78% contribution) differentiated the fungal communities of TC from those of RCC1, CC2, and CC3, and the second principal component (7.17% contribution) highlighted differences of the fungal communities in TC from those in RCC. Bray-Curtis distance matrix analysis indicated that fungal community composition differed between TC and RCC soils (Supplementary Fig. S1b).

According to the LEfSe, 3, 6, and 2 groups were enriched in TC, CC, and RCC soils, respectively (Supplementary Fig. S2b). These taxa were Trechisporales, Polyporales, Thysanoptera, Mortierellales, Tremellales, Rhizophlyctidales, Chaetothyriales, Hypnales, and Mucorales at the order level. The proportional distribution of fungal groups (>0.5%) varied at the order level (Fig. 5b). Compared with those of TC, the relative abundance of Pleosporales, Lecanorales, and Calosphaeriales increased, whereas those of Corticiaceae, Agaricales, and Tremellales decreased (except for CC3) in notoginseng cropping soils. The relative abundance of Capnodiales and Eurotiales showed an increasing trend in CC1, CC3, and RCC soils, whereas that of Verrucariales decreased by 29.9–38.6% in CC soils compared to those in TC soils. In addition, fungal groups with low relative abundance (<0.5%) fluctuated in CC and RCC soils compared to those in TC soils (Supplementary Table S4).

### Correlations between major bacterial groups and notoginseng death rates

**Figure 6:**
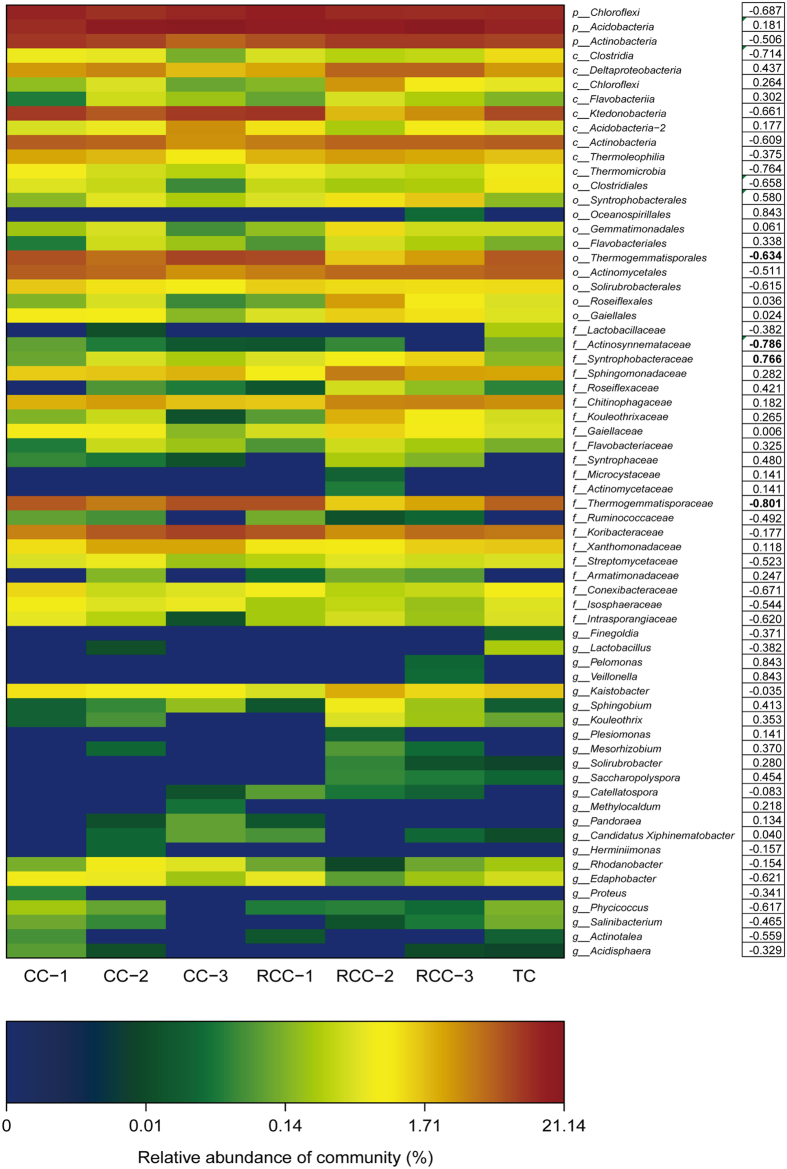
Relative abundance of the bacterial taxa detected by LEfSe as biomarker and their Pearson’s correlation coefficients with notoginseng death rates. Data are mean values of *n* = 3; significant correlation coefficients are noted in bold font where *P* < 0.05.

To determine the key microbial groups related to the death rates, we analyzed the relationships between bacterial taxa detected by LEfSe as biomarkers and notoginseng death rates (Fig. 6). Compared to those in TC, the relative abundance of Acidobacteria and Syntrophobacterales demonstrated increasing trends in those in CC and RCC, whereas the relative abundance of Clostridia, Clostridiales, and Actinosynnemataceae decreased in notoginseng cropping soils compared to those in TC soils. Pearson’s correlation analysis showed that Burkholderiales, and Syntrophobacteraceae were positively correlated with death rate (*P* < 0.05) (Fig. 6 and Supplementary Fig. S3). Thermogemmatisporales, Thermogemmatisporaceae, and Actinosynnemataceae were negatively related to notoginseng mortality (*P* < 0.05).

### Correlations between fungal taxa and notoginseng death rates

**Figure 7:**
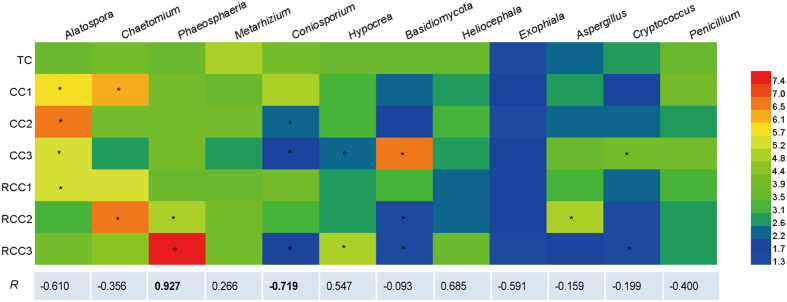
Relative abundance of the dominant genera of fungi (>1%) and their Pearson’s correlation coefficients with notoginseng death rates. Data are mean values of *n* = 3; asterisks denote significant differences between the TC and notoginseng cultivation in the relative abundance of fungal groups at *P* < 0.05. Significant correlation coefficients are noted in bold font where *P* < 0.05.

Several fungal genera were also related to notoginseng death rate (Fig. 7). Compared to those in TC, the relative abundance of *Phaeosphaeria* increased by 16.1–122% in CC and RCC soils, whereas the relative abundance of *Metarhizium*, *Coniosporium*, and *Heliocephala* decreased in notoginseng cropping soils. The relative abundance of *Alatospora* increased in CC and RCC1 soils compared to that in TC. The other fungal groups (relative abundance >1.0%) fluctuated in notoginseng cropping soils.

Pearson’s correlation analysis revealed that Hydnodontaceae, Herpotrichiellaceae, and *Coniosporium* were negatively related to death rate (*P* < 0.05), whereas *Myrmecridium*, *Phaeosphaeria*, *Phoma,* and *Fusarium* were positively related (*P* < 0.05) (Fig. 7; Supplementary Table S5; Supplementary Fig. S4).The positive correlation (*R* = 0.828, *P* < 0.05) between the relative abundance of *Fusarium* and the death rate was validated by qPCR analysis (Supplementary Fig. S5); this relationship was similar to that (*R* = 0.794, *P* < 0.05) between notoginseng mortality and the relative abundance of *Fusarium* that was observed from our high-throughput sequencing data.

The three fully identified species were *Coniosporium perforans*, *Phaeosphaeria rousseliana*, and *Fusarium oxysporum* in the genera of *Coniosporium*, *Phacosphaeria*, and *Fusarium,* respectively (Table 1). The relative abundance of *C. perforans* decreased by 13.1–60.0% (except for CC1), whereas the relative abundance of *F. oxysporum* increased by 22.7–263% (except for CC1) in notoginseng cultivation soils compared to those in TC. The relative abundance of *P. rousseliana* increased by 122% in RCC3 soils compared with that in TC. Pearson’s correlation analysis revealed that *C. perforans* was negatively related to death rates, and that *P. rousseliana* and *F. oxysporum* were positively related (*P* < 0.05) to death rate. The impact of a particular fungal strain (*F. oxysporum* PN-1) on seedling mortality was demonstrated by conducting pathogeny assays (Supplementary Fig. S6). The PN-1 strain was screened from the notoginseng cultivation soils and identified to be *F. oxysporum* on the basis of its morphology and molecular sequence. The symptoms of diseased notoginseng were discoloration or rot in the root, or dead seedlings. The notoginseng root-rot incidence and death rate were 71.3% and 6.0% after inoculation, respectively.

## Discussion

Seedling death is a serious problem in notoginseng CC systems, particularly in soils that have been previously planted with notoginseng, even after crop rotation. These problems hinder the development of perennial medicinal plants (e.g., *Rehmannia glutinosa*), which suffer from disease and reduced yield after the first year of cropping^7^. Severe reductions in the production of *Panax ginseng* have been caused by consecutive cultivation^22^, and the survival rate of ginseng has not exceeded 25% after three years^23^. Similar studies have reported that CC causes plant diseases, retarded growth, and yield losses^11^,^12^. Such crop losses result from diminished soil fertility, degradation of soil structure, and changes in the soil microorganism communities^6^,^24^,^25^. Changes in soil physicochemical properties are likely to cause changes in microbial community composition^8^,^9^. Our analyses did not show significant differences in pH, N, or organic matter between the soils of traditional cropping and notoginseng CC (Supplementary Table S6). Thus, we speculate that the cropping system was a dominant factor in disrupting the balance of soil microbial communities.

### Fungal diversity as a bioindicator of soil health status

Fungal diversity decreased in soils cropped continuously with notoginseng for three years compared to that of TC; however, bacterial diversity mainly showed an increasing trend under RCC. A previous study reported that the CC of peaches (*Prunus persica* L.) changed bacterial diversity in the soil^24^ but did not significantly affect the fungal diversity, as revealed by denaturing gradient gel electrophoresis^12^,^26^. These data suggested that the cropping system being applied affects microbial diversity.

Microbial diversity is critical for the maintenance of soil health and quality, indicating that it could be utilized as a sensitive bioindicator^27^,^28^. Microbial diversity and the suppression of root disease are closely related^29^,^30^. Reduced soil microbial diversity is responsible for the development of soil-borne diseases^31^. Fungal diversity helps to suppress plant diseases^32^. Therefore, the negative correlation between fungal diversity and death rate suggested that fungal diversity could serve as a bioindicator of soil health status in the notoginseng CC.

### Certain bacterial taxa were related to notoginseng death rates

Variation in bacterial community composition occurred under notoginseng cropping; this finding was consistent with those for other crops in which CC changed the composition of soil microorganisms^26^,^28^,^33^. The negative effects of CC increased with the number of years of pea cultivation, and severe changes in the soil environment also influenced pea production^6^.

Bacterial groups with low relative abundance (<0.6%) mainly decreased or were extinguished in CC soils compared with those in TC soils. Plant species affect microbial populations and could favor particular populations^9^. Notoginseng is a perennial plant, and its root exudates accumulate in the rhizosphere, providing substrates for several bacterial groups. However, not only do plants provide nutrients for microbial communities, but their root exudates also contain various antimicrobial metabolites^9^,^11^. This phenomenon might explain the reduction in the relative abundance of Bacteroidetes and Firmicutes, particularly in the CC system. Certain bacterial taxa antagonized plant pathogens, and their roles are as follows: fungistasis, antibiosis, modification of the biophysical root environment, active exclusion of the pathogenic fungi from the rhizosphere, and induction of plant disease resistance^34^. Negative relationships between death rate and bacterial taxa indicated that Thermogemmatisporaceae, and Actinosynnemataceae might be antagonists of pathogens. Our results offered references for the screening of beneficial agents for notoginseng.

### Fungi could act as pathogens or antagonists

Fluctuations in fungal community composition were described in notoginseng CC, and the findings were similar to those for other crops^12^. A previous study reported that with additional years of cropping, the relative abundance of Eurotiales, Glomerales, Hypocreales, and Tremellales increased, whereas that of Cantharellales, Agaricales, and Pezizales decreased^10^. Competition, antagonism, and hyperparasitism are the potential mechanisms contributing to complex changes in fungal communities^35^,^36^.

In our study, the positive correlations between death rates and fungal taxa suggested that *Myrmecridium*, *Fusarium*, *Phoma*, and *Phaeosphaeria* would be better adapted to colonization of diseased plants. *Fusarium* contains disease agents of notoginseng^21^; its relative abundance was positively associated with death rates (*P* < 0.05). The linear regression of the relationship (*R*^*2*^ = 0.891) between the relative abundance of *Fusarium* from high-throughput sequencing data and qPCR analysis indicated that the high-throughput sequencing data could offer key microbial taxa related to notoginseng mortality (Supplementary Fig. S7). *Fusarium oxysporum* is a severe root-rot pathogen^37^,^38^, and we confirmed the pathogenicity of notoginseng root-rot by using a pathogenic assay. The relationship between *F. oxysporum* and death rate revealed that the former is an important contributor to notoginseng mortality. *Phaeosphaeria* leaf spot caused severe damages to a growing area for maize (*Zea mays* L.) in eastern and southern Africa^39^. These data indicated that maize pathogens could be agents of soil-borne diseases. The relationship between *P. rousseliana* and death rates demonstrated that this species was associated with notoginseng mortality. In addition, a negative correlation revealed that *Coniosporium* could be a potential antagonist of notoginseng mortality.

In summary, our study presented the soil bacterial and fungal community dynamics in relation to notoginseng mortality in a CC system, and determined the pivotal microbial taxa related to death rates. The results provided information for the screening of beneficial microbes and pathogens of notoginseng. Our work would be of great significance for understanding CC obstacles caused by rhizospheric microorganisms.

## Materials and Methods

### Description of the experiment

This experiment was conducted in Yanshan, Yunnan Province (23°37′N, 104°20′E, 1300 m a.s.l.), which is the main production region for notoginseng. This region has an arid continental climate and laterite soils. The annual precipitation is 1000–1300 mm. Notoginseng was cultivated in strict accordance with the standard operating procedures established by the Good Agriculture Practices^40^,^41^.

Notoginseng is consecutively grown for three years before harvest in a fixed location. It is an ombrophyte, and the canopy coverage during its cultivation is 12–15% (Supplementary Fig. S8). A ridging cultivation pattern is used, with ridges approximately 1.4 m wide × 10 m long. Notoginseng gardens are commonly established in farmlands where maize plants are cultivated annually to provide a control system. The experiment was conducted as block design with three replicates, and the area of each replicated plot was 1.4 m × 10 m under the same management. Samples were obtained from plots planted with maize as the control. This experiment included six treatments: 1, 2, or 3 years of cultivation (CC1, CC2, and CC3); and 1, 2, or 3 years of replanting cultivation (RCC1, RCC2, and RCC3) after a 5-year rotation.

Our gardens were established in 2002, and the crops were cultivated in the gardens as described in the Supplementary Table S7. Among the plots in which notoginseng seedlings were planted, we replanted with notoginseng after a rotation, serving as an RCC treatment. Samples from those replanted plots continuously cultivated for 1, 2, and 3 years were designated as RCC1, RCC2, and RCC3, respectively. In addition, the plots first planted with notoginseng represented CC treatments, and samples from these plots continuously cultivated for 1, 2, and 3 years were designated as CC1, CC2, and CC3, respectively.

### Calculation of death rates and soil collection

An area (1.4 m × 2 m) was selected in each plot to calculate the death rates of notoginseng in each treatment. The mortality of notoginseng occurred from July to August, and the death rate of seedlings was surveyed in September of every year of notoginseng cultivation. The death rate was calculated for each plot as the number of dead seedlings divided by the total number of seedlings in the area. Three plots per treatment served as replicates.

Rhizosphere soil samples were collected according to Zhou and Wu^12^. Notoginseng seedlings were removed from plots, soil was shaken off, and rhizosphere fractions were brushed for further processing. Six seedlings of notoginseng or maize were selected randomly from each plot, and their soil samples were combined as a single sample. Soil samples were obtained from three replicates per treatment. In total, 21 soil samples were homogenized by being passed through a 2 mm sieve and stored at −80 °C until further processing. The soil characteristics are described in Supplementary Table S6.

### DNA extraction and quantitative PCR

Total soil DNA was extracted from 0.1 g of freeze-dried soil with the use of the MoBio Powersoil kit (MoBio Laboratories Inc., Carlsbad, CA) in accordance with the manufacturer’s instructions. DNA samples were stored at −20 °C until use. Quantitative PCR analysis was performed as previously described^16^. The relative abundances of bacterial small rRNA subunit genes were calculated using the Eub338/Eub518 primer pair as described previously^42^. The 5.8S/ITS1f primer pair was used to amplify the fungal gene fragments for the relative abundance of fungi^16^. Quantitative PCR was performed with the 2× SYBR Green PCR Master Mix (Takara Bio., Shiga, Japan) using an ABI7500 Fast Real-Time PCR system (Applied Biosystems, Foster City, CA, USA). Double-distilled water, rather than template DNA, was used as the control. Cycle threshold (*C*t) values were obtained from the known copy numbers in the standards. The ratio of fungi to bacteria was calculated based on the copy numbers.

### PCR amplification and Ion Torrent sequencing

For each sample, a 16S rRNA gene was amplified using conserved bacterial primers 27F/338R^43^. The 18S rRNA genes were amplified by conserved fungal primers 817F/1196R^16^. The forward and reverse primers contained a 10-bp barcode (Supplementary Table S8). Amplification reactions and purification were performed as previously described^43^. These amplicons were pooled in equimolar ratios. Sequencing was performed using an Ion Torrent Personal Genome Machine with an Ion Xpress Template kit (Life Technologies, Carlsbad, CA) and an Ion 314 chip (Life Technologies) following the manufacturer’s protocol.

### Processing of Ion Torrent sequences

Data were processed using the QIIME pipeline software^44^. Bacterial and fungal sequences were trimmed and assigned to different samples based on their barcodes. Sequences were binned into operational taxonomic units (OTUs) at the 97% similarity level. The representative sequence alignments were generated using PyNAST software. Sequence alignment was performed to remove gaps, and locations that were excessively variable were filtered^45^. The filtered alignment sequences were then used to build a phylogenetic tree using FastTree software^46^. The taxonomic identities of the bacteria and fungi were determined using RDP software^47^ and Silva schemes^48^. Beta-diversity metrics were used to assess the differences between the microbial communities, and an unweighted pair group method with an arithmetic mean tree was constructed from the full distance matrix.

Phylogenetic diversity statistics were computed for the Shannon index (*H*′), Chao1, and observed species modified according to Caporaso *et al*.^44^ In brief, QIIME randomly selected a series of subsets of each sample (75% of the smallest sample) for calculations, and this procedure was repeated 500 times for each dataset. Linear discriminant analysis (LDA) effect size (LEfSe) (http://huttenhower.sph.harvard.edu/lefse/) was used to characterize the features differentiating the microbial communities in soils as previously described^49^. In addition, certain species in the fungal genera related to death rates were identified by BLAST searches, and their similarity to the nearest relative was obtained in the National Center for Biotechnology Information (NCBI) database.

### Statistical analyses

SPSS version 11.0 software (SPSS Inc., Chicago, IL) was used for statistical analyses. These variables were considered for all treatment replicates and then subjected to ANOVA. Mean values were compared by calculating the least significant difference (LSD) at the 5% level in the analysis of death rates. Data were reported as significant or non-significant by paired *t*-tests (*P* < 0.05) in the analysis of the ratio of fungi to bacteria, microbial diversity, and relatively abundance in the TC system compared to notoginseng cultivation, respectively. Pearson’s correlation analyses were employed to correlate the death rates with the taxonomic diversity and relative abundance of the microbial communities.

## Additional Information

**Accession codes**: All metagenomic data have been submitted to the European Nucleotide Archive (http://www.ebi.ac.uk/): Accession number PRJEB4770 and PRJEB4773 for 16S rRNA genes and 18S rRNA genes,respectively.

**How to cite this article**: Dong, L. *et al*. Soil bacterial and fungal community dynamics in relation to *Panax notoginseng* death rate in a continuous cropping system. *Sci. Rep.* **6**, 31802; doi: 10.1038/srep31802 (2016).

## Supplementary Material

Supplementary Information

## Figures and Tables

**Table 1:** Identification and abundance of *Coniosporium perforans*, *Fusarium oxysporum*, and *Phaeosphaeria rousseliana* from the fungal operational taxonomic units (OTUs).

Closest database match	Closest accession number	Similarity (%)	Relative abundance (%)							*R*
			TC	CC1	CC2	CC3	RCC1	RCC2	RCC3	
*Coniosporium perforans*	AJ972863.1	100	2.14	2.76*	1.20*	1.37*	1.32*	1.86	0.86*	−0.575
*Fusarium oxysporum*	JF807402.1	98	0.07	0.05	0.16*	0.19*	0.09	0.09	0.25*	**0.841**
*Phaeosphaeria rousseliana*	FN666096.1	100	2.52	2.44	2.57	2.02	2.31	2.86	5.61*	**0.830**

*R* stands for the relationship between the relative abundance of three fungal species and notoginseng death rate based on the Pearson’s correlation analysis. Data are presented as means of *n* = 3. Asterisks denote significant differences between the TC and notoginseng cultivation in the relative abundance of fungal groups at *P* < 0.05. Significant correlation coefficients are noted in bold font where *P* < 0.05.
