# Supplementary material for: Soil bacterial and fungal community dynamics in relation to Panax notoginseng death rate in a continuous cropping system
Source: Sci Rep. 2016 Aug 23;6:31802. doi: 10.1038/srep31802 (PMC4994099; doi:10.1038/srep31802)
Supplement: Supplementary Information [file srep31802-s1.pdf]

Soil bacterial and fungal community dynamics in relation to *Panax notoginseng* death rate in a continuous cropping system

Linlin Dong[1], Jiang Xu[1], Guangquan Feng[2], Xiwen Li[1], Shilin Chen[1*]

[1] Institute of Chinese Materia Medica, China Academy of Chinese Medical Sciences, Beijing 100700, China.

[2] Wenshan Prefecture Sanqi Science and Technology Research Institute, Sanqi Research department, Yunnan province 663000, China.

* Correspondence author: Shilin Chen

Tel: 0086-10- 57203877; Fax: 0086-10- 62899776; E-mail: slchen@icmm.ac.cn

Materials and methods

Separation and identification of pathogenic strains

Soil samples (1 g) from the rhizosphere of notoginseng infected with root rot were diluted 1000 times, and the samples (100 μL) were used to screen pathogenic strains using acidifier potato glucose medium (APDA)[1]. The separated and purified strains were inoculated on the APDA at 25 °C for 3 d for identification.

Morphological identification was confirmed according to the description of Wei[1]. Molecular identification of the selected strain was performed by the amplification of 18S rRNA with universal primers NS1 and NS6[2]. Genomic DNA of a single enriched strain was extracted using the cetyl trimethyl ammonium bromide (CTAB) method[3]. PCR was conducted in a 25 μL reaction mixture with *Taq* DNA recombinant polymerase (TaKaRa Bio.). Negative controls (no template DNA) were included to check for primer and sample DNA contamination. The sequences were analyzed on a 3730 XL sequencer (Applied Biosystems, Foster city, CA, USA). The generated sequence was compared with published 18S rRNA sequences on the National Center for Biotechnology Information web site using the BLAST query search engine (http://www.ncbi.nlm.gov/blast). The sequences produced in this study have been submitted to GenBank with accession numbers (KX086739). Neighbor-joining (NJ) trees were constructed in MEGA v6.0 software to generate Kimura 2-parameter (K2P) distance matrices for each sequence following standard parameters.

Supplementary information

Pathogenicity assays

Pathogenicity of the identified strains was tested using pot experiments in our plantation. Three years of notoginseng seedlings were cultivated in the pots containing 1,000 g of heat-sterilized soil. The 10 mL cultures (2.0×10^6 cfu mL^{-1}) of strains were inoculated into the pots after transplanting for one month. The inactivated cultures were also inoculated as the control. Fifty replicate seedlings were used. The symptoms of notoginseng seedlings were analyzed after inoculation for 45 d. Disease severity was assessed with a 0 to 3 visual scale, where 0=no symptoms, 1=light or moderate discoloration in the root, 2=severe discoloration or rot in the root, and 3=dead seedlings. The numbers of diseased seedlings were calculated according to the disease severity. To confirm the infection of notoginseng plants by strains, isolations were made from a diseased plant with symptoms.

Quantitative PCR of pathogenic taxa

To verify the relationship between the relative abundance of key pathogenic taxa and notoginseng death rate, the relative abundance of *Fusarium* was calculated using the ITS-Fu-F/ITS-Fu-R[4] by quantitative PCR according to the description of Rousk *et al.*[5].

Fig. S1

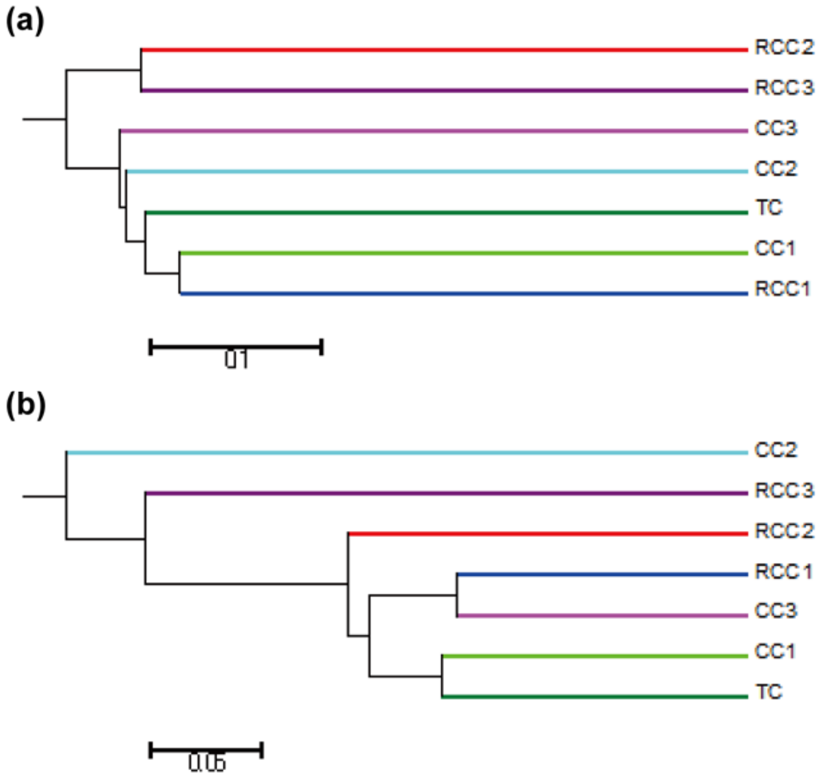

Fig. S1 Dendrogram of microbial communities in notoginseng cropping and traditional cropping soils. a) Clustering showed the relatedness of samples that were separated using Bray-Curtis distances of classified 16S rRNA, and **b)** 18S rRNA gene sequences, respectively. TC, traditional cropping; CC1, CC2 and CC3 indicated continuous cropping for 1, 2 and 3 years, respectively; RCC1, RCC2 and RCC3 represented replanted continuous cropping systems for 1, 2 and 3 years, respectively. Data were means of $n = 3$.

Fig.S2

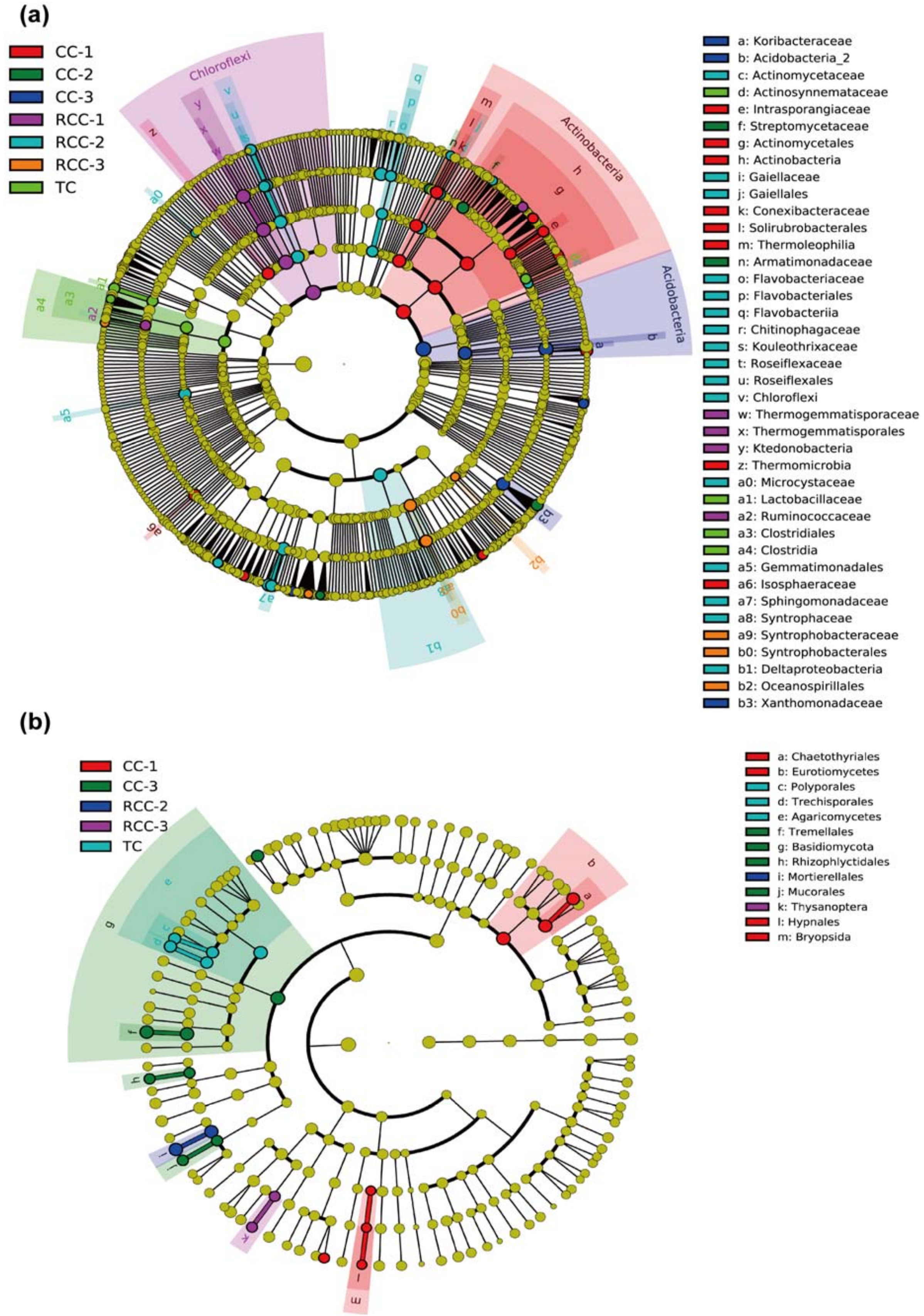
(a)
CC-1
CC-2
CC-3
RCC-1
RCC-2
RCC-3
TC
Chloroflexi
Actinobacteria
Acidobacteria
a: Koribacteraceae
b: Acidobacteria_2
c: Actinomycetaceae
d: Actinosynnemataceae
e: Intrasporangiaceae
f: Streptomycetaceae
g: Actinomycetales
h: Actinobacteria
i: Gaiellaceae
j: Gaiellales
k: Conexibacteraceae
l: Solirubrobacterales
m: Thermoleophilia
n: Armatimonadaceae
o: Flavobacteriaceae
p: Flavobacteriales
q: Flavobacteriia
r: Chitinophagaceae
s: Kouleothrixaceae
t: Roseiflexaceae
u: Roseiflexales
v: Chloroflexi
w: Thermogemmatisporaceae
x: Thermogemmatisporales
y: Ktedonobacteria
z: Thermomicrobia
a0: Microcystaceae
a1: Lactobacillaceae
a2: Ruminococcaceae
a3: Clostridiales
a4: Clostridia
a5: Gemmatimonadales
a6: Isosphaeraceae
a7: Sphingomonadaceae
a8: Syntrophaceae
a9: Syntrophobacteraceae
b0: Syntrophobacterales
b1: Deltaproteobacteria
b2: Oceanospirillales
b3: Xanthomonadaceae
(b)
CC-1
CC-3
RCC-2
RCC-3
TC
a: Chaetothyriales
b: Eurotiomycetes
c: Polyporales
d: Trechisporales
e: Agaricomycetes
f: Tremellales
g: Basidiomycota
h: Rhizophlyctidales
i: Mortierellales
j: Mucorales
k: Thysanoptera
l: Hypnales
m: Bryopsida

Fig.S2 LEfSe results on soil microbial communities. a) Taxonomic cladogram obtained from LEfSe of 16S OTU. **b)** Taxonomic cladogram obtained from LEfSe of 18S OTU. The cladograms reported the taxa (highlighted by small circles and shading) showing different abundance values in the soils of TC, CC and RCC. Yellow circles represented non-significant differences in abundance between samples of those particular taxa. Each circle's diameter was proportional to the taxon's abundance. TC indicated traditional cropping (control). CC1, CC2 and CC3 indicated continuous cropping for 1, 2 and 3 years, respectively; RCC1, RCC2 and RCC3 represented replanted continuous cropping systems for 1, 2 and 3 years, respectively. Data were means of $n = 3$.

Fig.S3

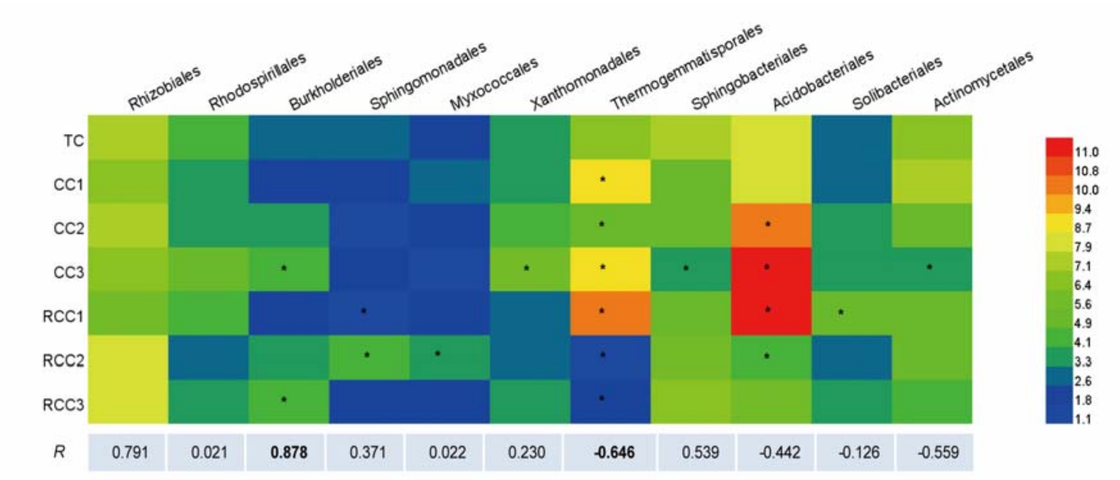

Fig.S3 The relative abundance of the dominant bacterial taxa (>1%) and their Pearson's correlation coefficients with notoginseng death rates. TC indicated traditional cropping (control). CC1, CC2 and CC3 indicated continuous cropping for 1, 2 and 3 years, respectively; RCC1, RCC2 and RCC3 represented replanted continuous cropping systems for 1, 2 and 3 years, respectively. Data were means of $n = 3$; asterisks denoted significant differences between the TC and notoginseng cultivation in the relative abundance of soil bacterial groups at $P < 0.05$. Significant correlation coefficients were noted in bold font where $P < 0.05$.

Fig.S4

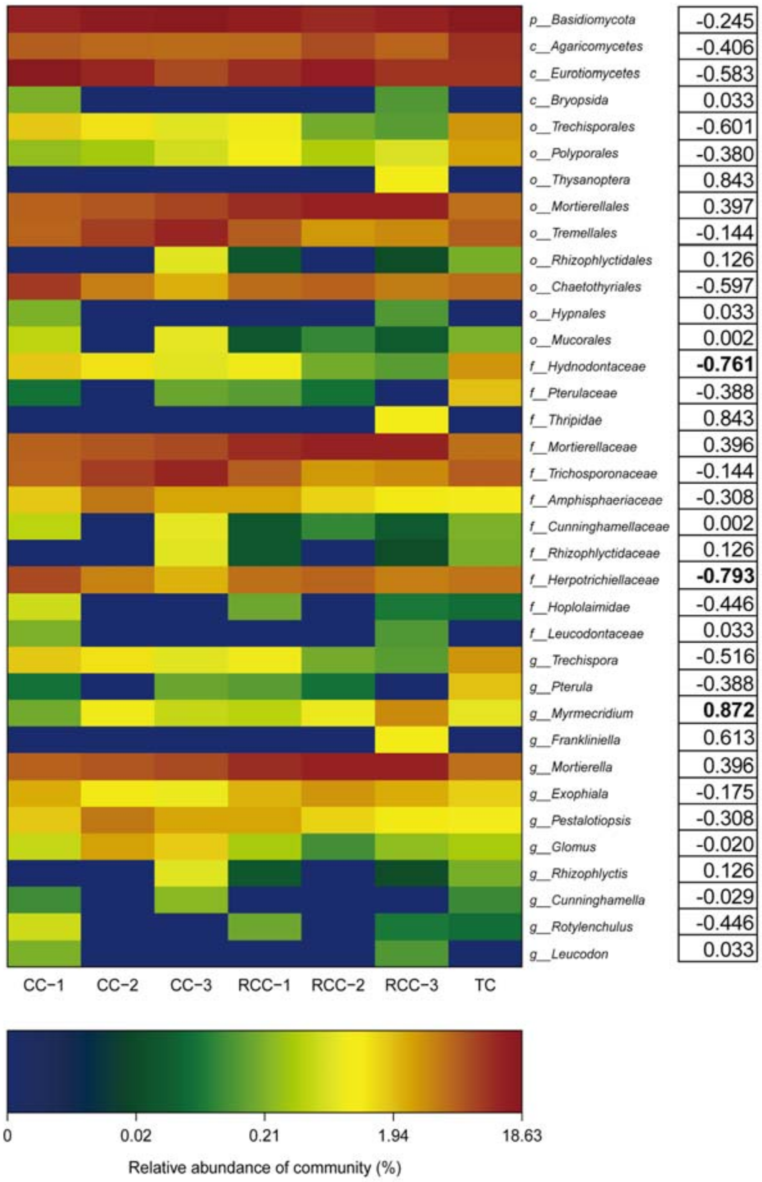

Fig.S4 The relative abundance of the fungal taxa detected by LEfSe as biomarkers and their Pearson's correlation coefficients with notoginseng death rates. TC indicated traditional cropping (control). CC1, CC2 and CC3 indicated continuous cropping for 1, 2 and 3 years, respectively; RCC1, RCC2 and RCC3 represented replanted continuous cropping systems for 1, 2 and 3 years, respectively. Data were mean values of n = 3; significant correlation coefficients were noted in bold font where $P < 0.05$.

Fig.S5

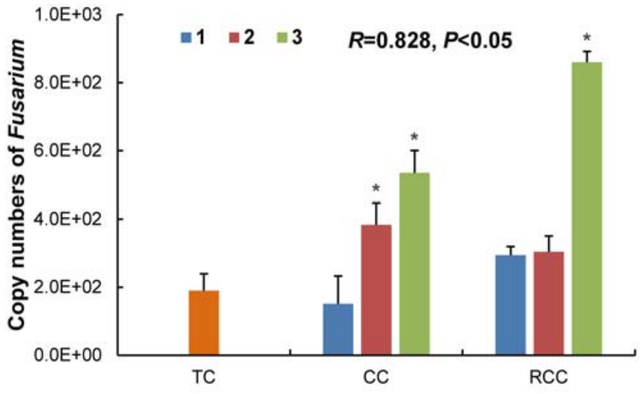

Fig.S5 The abundance of *Fusarium* in the soils. TC indicated traditional cropping (control). CC and RCC indicated continuous cropping and replanted continuous cropping, respectively. *R* presented the relationship between the abundance of *Fusarium* and notoginseng death rates based on the Pearson's correlation analysis. All values were indicated as the mean ± SE (n = 3); asterisks denoted significant differences between the traditional cropping and notoginseng cultivation in the abundance of *Fusarium* at $P < 0.05$.

Fig. S6

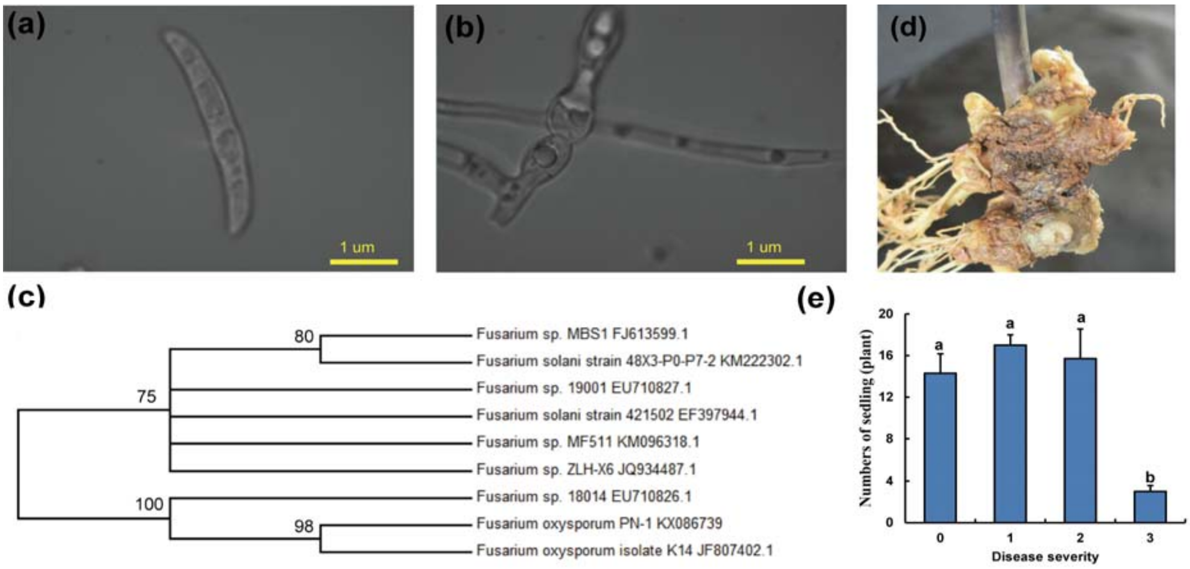

Fig. S6 The pathogenicity analysis of strains screened from soil. a) Conidium and **b)** chamydospore of notoginseng root-knot pathogenic strain. **c)** The relationships among strains (KX086739) and published 18S rDNA sequences (GenBank accession numbers were presented in parentheses). **d)** The symptoms of root-rot caused by separated strain in the notoginseng (There were no symptoms of notoginseng root-rot in the control). **e)** The ratio of disease severity in the notoginseng after inoculation. Neighbor-joining (NJ) trees were constructed in MEGA v6.0 to generate Kimura 2-parameter (K2P) distance matrices for each sequence following standard parameters. The numbers at the branch knots were bootstrap values based on 1000 resamplings for the maximum likelihood. Only bootstrap values greater than 75% were shown. All values were indicated as the mean ± SE (n = 3). Bars with different letters denoted significant differences at $\alpha = 0.05$. 0, 1, 2, and 3 represented no symptoms, light or moderate discoloration in the root, severe discoloration or rot in the root, and dead seedlings after inoculation.

Fig.S7

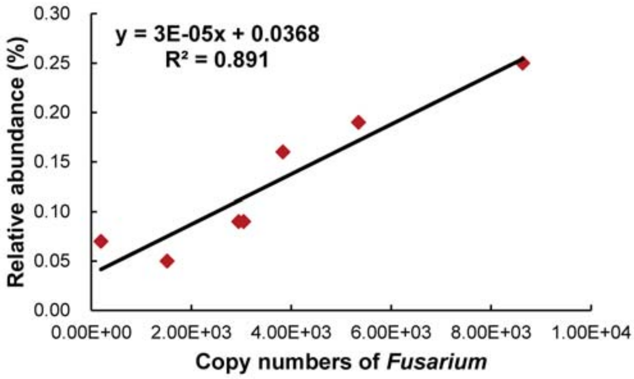

Fig. S7 Linear regression of the relationship between relative abundance (%) of *Fusarium* from high-throughput sequencing data and qPCR analysis. All values were indicated as the mean of $n = 3$.

Fig. S8

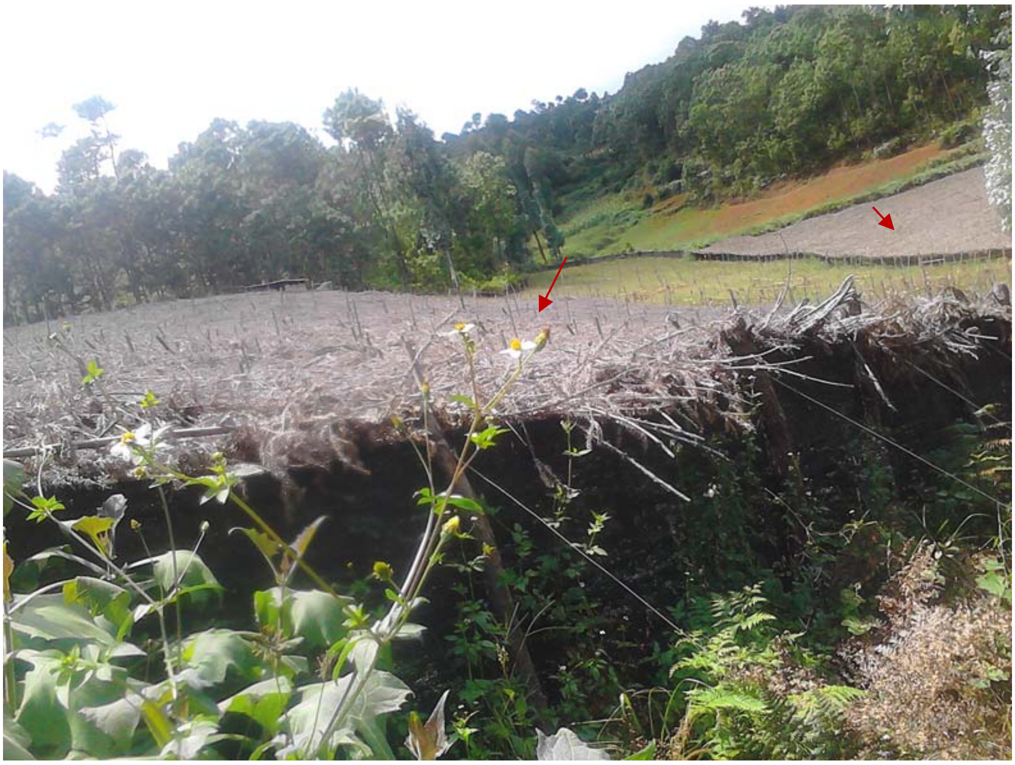

Fig. S8 The cultivation pattern of notoginseng in China. Red arrows represented notoginseng field station.

Table S1. The death rates (%) of notoginseng seedlings every year.

Cropping systems	2002	2003	2004	2005	2006	2010	2011	2012
TC	-	-	-	-	-	-	-	-
CC1	-	-	-	-	-	-	-	**2.0**
CC2	-	-	-	-	-	-	**1.5**	**14.4**
CC3	-	-	-	-	-	**1.3**	**15.2**	**34.3**
RCC1	-	-	**3.4**	**16.9**	**39.7**	-	-	**2.6**
RCC2	-	**1.8**	**8.6**	**29.2**	-	-	**2.7**	**39.4**
RCC3	**2.1**	**14.2**	**35.2**	-	-	**3.8**	**35.2**	**81.2**

TC indicated traditional cropping (control); CC1, CC2 and CC3 indicated continuous cropping for 1, 2 and 3 years, respectively; RCC1, RCC2 and RCC3 represented replanted continuous cropping for 1, 2 and 3 years, respectively. – indicated no data. Data were presented as the mean of $n = 3$.

Table S2. Numbers of bacterial and fungal sequences, derived OTUs and average length in each sample

Samples	Bacterial community			Fungal community		
	Sequences	OTUs	Average Length	Sequences	OTUs	Average Length
TC1	1849	1416	220	2379	855	243
TC2	2852	1960	220	1224	560	225
TC3	2197	1604	221	1073	476	234
CC1-1	998	794	231	596	323	227
CC1-2	1358	1042	226	1610	593	238
CC1-3	970	807	209	1314	518	247
CC2-1	2097	1656	219	300	185	220
CC2-2	2284	1716	219	507	287	231
CC2-3	988	805	205	737	405	238
CC3-1	1552	1167	216	958	404	232
CC3-2	1328	1030	211	1747	638	243
CC3-3	1941	1466	222	1576	629	245
RCC1-1	917	745	218	481	304	226
RCC1-2	1432	1131	215	1242	536	228
RCC1-3	855	712	220	948	494	240
RCC2-1	2840	2061	224	871	455	222
RCC2-2	2157	1695	225	2089	696	254
RCC2-3	2079	1652	222	1417	643	247
RCC3-1	2447	1841	219	3374	969	245
RCC3-2	2325	1752	217	2554	768	241
RCC3-3	2906	2101	206	2418	787	231

TC indicated traditional cropping (control); CC1, CC2 and CC3 indicated continuous cropping for 1, 2 and 3 years, respectively; RCC1, RCC2 and RCC3 represented replanted continuous cropping for 1, 2 and 3 years, respectively. -1,-2 and -3 presented three replicates.

Table S3. Relative abundances (%) of rare bacterial phyla

Phyla	TC	CC1	CC2	CC3	RCC1	RCC2	RCC3
WPS-2	1.34±012	1.22±0.26	0.74±0.23*	0.95±0.12*	1.29±0.21	0.12±0.23*	0.22±0.12*
TM7	0.80±0.13	0.78±0.13	1.14±0.15	0.41±0.07*	0.31±0.12*	0.56±0.13	0.70±0.23
Armatimonadetes	0.92±0.14	0.72±0.16	0.81±0.10	0.45±0.03*	0.69±0.15	1.15±0.10	0.94±0.22
Nitrospirae	0.48±0.12	0.36±0.09	0.59±0.12	0.76±0.15	0.55±0.11	0.77±0.04*	0.74±0.04*
Cyanobacteria	1.04±0.27	0.58±0.13	1.58±0.28	1.31±0.19	1.02±0.16	1.15±0.02	1.67±0.22*
Chlorobi	0.27±0.11	0.15±0.04	0.19±0.02	0.14±0.07	0.08±0.04*	0.08±0.03*	0.19±0.08
Fibrobacteres	0.07±0.01	0.06±0.03	0.04±0.03	0.00±0.00*	0.00±0.00*	0.05±0.02	0.00±0.00*
TM6	0.29±0.08	0.64±0.18*	0.40±.011	0.31±0.10	0.20±0.03	0.30±0.10	0.17±0.08
Tenericutes	0.15±0.09	0.08±0.04	0.06±0.02	0.06±0.01	0.02±0.02	0.14±0.08	0.12±0.08
Elusimicrobia	0.48±0.17	0.47±0.11	0.25±0.14	0.19±0.09	0.25±0.09	0.25±0.10	0.37±0.05

TC indicated traditional cropping (control); CC1, CC2 and CC3 indicated continuous cropping for 1, 2 and 3 years, respectively; RCC1, RCC2 and RCC3 represented replanted continuous cropping for 1, 2 and 3 years, respectively. Their average relative abundances were less than 0.6% in the control and treatments. Data were presented as the mean ± SE of n = 3, and asterisks denoted significant differences between the traditional cropping and notoginseng cultivation in the relative abundance of bacterial groups at $P < 0.05$.

Table S4. Relative abundances of the major taxa of rare fungal groups at the order level

Taxa	TC	CC1	CC2	CC3	RCC1	RCC2	RCC3
Blastocladiales	0.72±0.20	0.58±0.26	0.25±0.16*	0.50±0.04	0.33±0.17	0.51±0.21	0.36±0.08*
Onygenales	0.26±0.14	0.65±0.08*	0.42±0.14	0.37±0.08	0.37±0.08	0.61±0.18	0.74±0.22*
Ostropales	0.33±0.15	0.39±0.07	0.11±0.06*	0.10±0.05*	0.26±0.15	0.16±0.03	0.08±0.00*
Helotiales	1.33±0.13	2.69±0.67*	3.36±1.18*	2.51±0.53*	2.63±1.22	0.00±0.00*	0.00±0.00*
Meliolales	0.55±0.07	0.51±0.14	0.18±0.12*	0.57±0.03	0.21±0.11*	0.40±0.09	0.45±0.07
Boletales	1.95±0.61	0.50±0.18*	0.36±0.20*	0.57±0.06*	0.50±0.13*	0.44±0.16*	0.30±0.18*
Mucorales	0.89±0.44	1.06±0.11	0.83±0.47	1.56±0.71	0.87±0.10	0.38±0.08	0.51±0.25
Glomerales	0.22±0.05	0.09±0.05	0.51±0.32	0.16±0.09	0.30±0.27	0.46±0.22	0.49±0.26
Cystofilobasidiales	0.47±0.20	0.55±0.22	0.63±0.12	1.05±0.25	0.71±0.35	0.48±0.07	1.83±1.43
Kickxellales	0.22±0.09	0.13±0.07	0.16±0.08	0.08±0.06	0.32±0.06	0.29±0.09	0.35±0.17
Pyxidiophorales	0.20±0.07	0.10±0.10	0.42±0.14	0.14±0.08	0.12±0.07	0.17±0.13	0.13±0.06
Orbiliales	0.18±0.06	0.24±0.04	0.13±0.13	0.21±0.02	0.21±0.21	0.35±0.28	0.12±0.08
Magnaporthales	0.16±0.02	0.10±0.03	0.27±0.04*	0.10±0.05	0.16±0.03	0.18±0.10	0.20±0.02
Ophiostomatales	0.10±0.06	0.05±0.02	0.31±0.19	0.02±0.02	0.14±0.07	0.09±0.09	0.11±0.02
Mortierellales	0.19±0.09	0.47±0.08	0.11±0.11	0.29±0.22	0.19±0.02	0.17±0.09	0.21±0.05
Chytridiales	0.06±0.03	0.05±0.05	0.11±0.11	0.19±0.09	0.07±0.06	0.13±0.07	0.05±0.05
Dothideales	0.14±0.05	0.05±0.02*	0.11±0.11	0.15±0.03	0.06±0.03	0.09±0.06	0.09±0.02
Sporidiobolales	0.23±0.13	0.08±0.05	0.20±0.14	0.18±0.07	0.27±0.11	0.16±0.09	0.11±0.04
Microbotryales	0.18±0.06	0.08±0.05	0.09±0.09	0.17±0.14	0.31±0.18	0.12±0.07	0.09±0.09
Sebacinales	0.04±0.04	0.02±0.02	0.20±0.11	0.04±0.04	0.04±0.03	0.23±0.10*	0.00±0.00
Erysiphales	0.13±0.02	0.07±0.04	0.07±0.06	0.10±0.08	0.17±0.13	0.00±0.00*	0.06±0.04

TC indicated traditional cropping; CC1, CC2 and CC3, respectively indicated continuous cropping for 1, 2 and 3 years; RCC1, RCC2 and RCC3 represented replanted continuous cropping for 1, 2 and 3 years, respectively, after rotation. Their average relative abundances were less than 0.5% in the control and treatments. Data were presented as the mean ± SE of $n = 3$, and asterisks denoted significant differences between the traditional cropping and notoginseng cultivation in the relative abundance of fungal groups at $P < 0.05$.

Table S5. The relative abundance of *Fusarium* and *Phoma*, and their Pearson's correlation coefficients with notoginseng death rates

Treatments	*Fusarium*	*Phoma*
TC	0.49±0.03	0.19±0.01
CC1	0.49±0.02	0.18±0.02
CC2	0.60±0.01*	0.16±0.01
CC3	0.70±0.02*	0.18±0.03
RCC1	0.51±0.03	0.16±0.01
RCC2	0.44±0.02	0.17±0.02
RCC3	0.83±0.03*	0.25±0.02*
R	**0.794**	**0.875**

CC1, CC2 and CC3 indicated continuous cropping for 1, 2 and 3 years, respectively ; RCC1, RCC2 and RCC3 represented replanted continuous cropping for 1, 2 and 3 years, respectively. Data were presented as the mean ± SE of $n = 3$; asterisks denoted significant differences between the traditional cropping and notoginseng cultivation in the relative abundance of fungal genera at $P < 0.05$. Black body denoted significant differences between the genera and notoginseng mortality at $P < 0.05$ based on the Pearson's correlation analysis.

Table S6. Soil chemical characteristics in soils of traditional cropping and notoginseng continuous cropping

Treatments	pH	Total N ($g\ kg^{-1}$)	Olsen-P ($g\ kg^{-1}$)	K ($mg\ kg^{-1}$)	Organic matter ($g\ kg^{-1}$)
TC	5.86±0.11	1.50±0.02	33.02±5.51	272.08±3.96	20.96±4.98
CC1	5.36±0.09	1.27±0.06	48.81±2.89	167.91±9.72*	19.87±0.56
CC2	5.57±0.06	1.13±0.01	46.10±2.87	351.20±18.46	22.63±1.38
CC3	5.26±0.09	1.50±0.06	61.13±3.00*	146.81±5.49*	32.25±2.87
RCC1	5.40±0.13	1.23±0.01	51.88±2.07	190.33±8.78*	25.42±0.88
RCC2	5.34±0.11	1.13±0.04	39.50±4.98	258.90±17.21	26.57±1.84
RCC3	5.65±0.01	1.48±0.01	36.08±3.63	326.15±14.10	29.26±0.83

TC indicated traditional cropping (control); CC1, CC2 and CC3 indicated continuous cropping for 1, 2 and 3 years respectively; RCC1, RCC2 and RCC3 represented replanted continuous cropping for 1, 2 and 3 years, respectively. Data were presented as the mean ± SE of $n = 3$, and asterisks indicated significant differences between the traditional cropping and notoginseng cultivation in the soil chemical characteristics at $P < 0.05$.

Table S7. The crops were cultivated in the notoginseng garden from 2002 to 2012

Cropping systems	2002	2003	2004	2005	2006	2007	2008	2009	2010	2011	2012
TC	M	M	M	M	M	M	M	M	M	M	M
CC1	M	M	M	M	M	M	M	M	M	M	**N**
CC2	M	M	M	M	M	M	M	M	M	**N**	**N**
CC3	M	M	M	M	M	M	M	M	**N**	**N**	**N**
RCC1	M	M	**N**	**N**	**N**	M	M	**HP**	M	M	**N**
RCC2	M	**N**	**N**	**N**	M	M	**HP**	M	M	**N**	**N**
RCC3	**N**	**N**	**N**	M	M	**HP**	M	M	**N**	**N**	**N**

TC: Traditional cropping; CC1, CC2 and CC3: continuous cropping for 1, 2 and 3 years; RCC1, RCC2 and RCC3: replanting continuous cropping for 1, 2 and 3 years. M: maize; N: notoginseng; HP: hot pepper.

Table S8. The 10-bp barcodes used to tag each analyzed PCR product

ACGTTGAATC	AGTAGTGATC	ATGTACGATG
ACTAGCAGTA	AGTGTATGTC	ATGTGTCTAG
ACTCATCTAC	AGTTCAAGTC	CAGTTCAAGT
ACTTGTTCAG	AGTTCTTGAC	CATACTCTAC
AGCTTCTTAG	ATACGACGTA	CATAGTAGTG
AGCTTCTTGA	ATATAGTCGC	CATTGAAGCT
AGTACGCTAT	ATCTACTGAC	CATTGTTAGC

Bacterial and fungal products were sequenced in different runs, and these 21 barcodes were used respectively for distinguishing the bacterial and fungal samples.

References

1. Wei, J.C. Fungal identification manual. Shanghai: Shanghai Scientific and Technical Publishers (1979).
2. Lin, X.M., Li, Z.Q., Wang, S.X. The characteristics of rDNA in Fungi and its using for identification of ectomycorrhizal fungi. *Acta. Agri. Boreali-occidentalis Sinica*. **14**,120–125 (2005).
3. He, Y.T., Yang, R.D., Zhang, G., Huang, Y.W. Identification of a cellulose-producing fungi based on 18S rDNA sequence. *Modern food Sci. Technol.* **24**, 638–640 (2008).
4. Abd-Elsalam, K.A., Aly, I.N., Abdel-Satar, M.A., Khalil, M.S., Verreet, J.A. PCR identification of Fusarium genus based on nuclear ribosomal-DNA sequence data. *Afr. J. Biotechnol.* **2**, 82–85 (2003).
5. Rousk, J. *et al*. Soil bacterial and fungal communities across a pH gradient in an arable soil. *ISME J.* **4**, 1–12 (2010).
